# Variation in revascularisation use and outcomes of patients in hospital with acute myocardial infarction across six high income countries: cross sectional cohort study

**DOI:** 10.1136/bmj-2021-069164

**Published:** 2022-05-04

**Authors:** Peter Cram, Laura A Hatfield, Pieter Bakx, Amitava Banerjee, Christina Fu, Michal Gordon, Renaud Heine, Nicole Huang, Dennis Ko, Lisa M Lix, Victor Novack, Laura Pasea, Feng Qiu, Therese A Stukel, Carin Uyl de Groot, Lin Yan, Bruce Landon

**Affiliations:** 1 Department of Medicine, University of Texas Medical Branch, Galveston, TX, USA; 2 ICES, Toronto, ON, Canada; 3 Faculty of Medicine, University of Toronto, Toronto, ON, Canada; 4 Department of Health Care Policy, Harvard Medical School, Boston, MA, USA; 5 Erasmus School of Health Policy and Management, Erasmus University, Rotterdam, Netherlands; 6 Institute of Health Informatics, University College London, London, UK; 7 Department of Cardiology, University College London Hospitals, London, UK; 8 Clinical Research Center, Soroka University Medical Center, Faculty of Health Sciences, Ben Gurion University of the Negev, Beersheba, Israel; 9 Institute of Hospital and Health Care Administration, National Yang-Ming Chiao Tung University, Taipei, Taiwan; 10 Schulich Heart Research Institute, Sunnybrook Health Sciences Centre, Toronto, ON, Canada; 11 Department of Community Health Sciences, University of Manitoba, Winnipeg, MB, Canada; 12 Institute of Health Policy, Management and Evaluation, University of Toronto, Toronto, ON, Canada; 13 George & Fay Yee Centre for Healthcare Innovation, University of Manitoba, Winnipeg, MB, Canada; 14 Division of General Medicine, Beth Israel Deaconess Medical Center, Boston, MA, USA

## Abstract

**Objectives:**

To compare treatment and outcomes for patients admitted to hospital with a primary diagnosis of ST elevation or non-ST elevation myocardial infarction (STEMI or NSTEMI) in six high income countries with very different healthcare delivery systems.

**Design:**

Retrospective cross sectional cohort study.

**Setting:**

Patient level administrative data from the United States, Canada (Ontario and Manitoba), England, the Netherlands, Israel, and Taiwan.

**Participants:**

Adults aged 66 years and older admitted to hospital with STEMI or NSTEMI between 1 January 2011 and 31 December 2017.

**Outcomes measures:**

The three categories of outcomes were coronary revascularisation (percutaneous coronary intervention or coronary artery bypass graft surgery), mortality, and efficiency (hospital length of stay and 30 day readmission). Rates were standardised to the age and sex distribution of the US acute myocardial infarction population in 2017. Outcomes were assessed separately for STEMI and NSTEMI. Performance was evaluated longitudinally (over time) and cross sectionally (between countries).

**Results:**

The total number of hospital admissions ranged from 19 043 in Israel to 1 064 099 in the US. Large differences were found between countries for all outcomes. For example, the proportion of patients admitted to hospital with STEMI who received percutaneous coronary intervention in hospital during 2017 ranged from 36.9% (England) to 78.6% (Canada; 71.8% in the US); use of percutaneous coronary intervention for STEMI increased in all countries between 2011 and 2017, with particularly large rises in Israel (48.4-65.9%) and Taiwan (49.4-70.2%). The proportion of patients with NSTEMI who underwent coronary artery bypass graft surgery within 90 days of admission during 2017 was lowest in the Netherlands (3.5%) and highest in the US (11.7%). Death within one year of admission for STEMI in 2017 ranged from 18.9% (Netherlands) to 27.8% (US) and 32.3% (Taiwan). Mean hospital length of stay in 2017 for STEMI was lowest in the Netherlands and the US (5.0 and 5.1 days) and highest in Taiwan (8.5 days); 30 day readmission for STEMI was lowest in Taiwan (11.7%) and the US (12.2%) and highest in England (23.1%).

**Conclusions:**

In an analysis of myocardial infarction in six high income countries, all countries had areas of high performance, but no country excelled in all three domains. Our findings suggest that countries could learn from each other by using international comparisons of patient level nationally representative data.

## Introduction

Interest is growing in the use of international comparisons to gain a deeper understanding of the tradeoffs inherent in the healthcare systems of different countries.^1^ These comparisons can reveal opportunities for improvement that are difficult to identify through within country analyses alone.^1^ ^2^ Many international comparisons have used aggregated data from the World Health Organization or the Organisation for Economic Cooperation and Development (OECD) and revealed that the United States spends more on healthcare and has worse outcomes than other high income countries.^3^ ^4^ ^5^ However, these studies typically lack the granularity to understand why certain countries appear to perform better and why others fall short.^6^

We established the International Health Systems Research Collaborative (IHSRC: https://projects.iq.harvard.edu/ihsrc/people) to enable comparisons of high income countries (the US, Canada, England, Netherlands, Israel, and Taiwan) using a different approach. The IHSRC uses nationally representative patient level data from participating countries to identify patients with the same diagnoses or undergoing the same procedures to compare processes of care, outcomes, and measures of efficiency across countries.^6^ ^7^ ^8^ The six IHSRC countries were chosen because all have highly developed healthcare systems and accessible administrative data, but differ in their financing, organisation, and overall performance in international rankings.^3^ ^9^

We selected acute myocardial infarction (AMI) for our initial study because it is an ideal condition for cross country comparisons. AMI is a common condition with established international diagnostic criteria and consensus about evidence based treatments, and has well developed coding schemes for identification using administrative data.^10^ ^11^ ^12^ ^13^ Additionally, hospital admission is the standard of care for AMI in high income countries, which minimises potential selection bias. A small body of literature compares AMI across high income countries, but existing studies are outdated,^14^ ^15^ do not include the US or Canada,^8^ ^16^ use aggregated population level or hospital level data rather than patient level data,^17^ or have not undergone peer review.^13^ ^18^ Moreover, most studies have failed to differentiate ST elevation and non-ST elevation myocardial infarction (STEMI and NSTEMI) despite important differences in how these conditions are managed (early percutaneous coronary intervention (PCI) for STEMI).^15^ ^17^ ^18^ ^19^

The objective of our study was to quantify differences between countries in treatment, outcomes, and measures of efficiency for patients admitted to hospital with STEMI or NSTEMI between 2011 and 2017 in six IHSRC countries. We anticipated that all countries would have areas of high performance and low performance, but that no country would excel across all measures.

## Methods

### Data and patients

We conducted a retrospective cohort study of adults aged 66 years and older admitted to hospital for one day or longer (but including those who died on the day of admission) with a primary diagnosis of AMI between 1 January 2011 and 31 December 2017 (2013-17 for the Netherlands) in the six IHSRC countries. We used nationally representative administrative data from each country (described in detail in supplementary appendix 1). Patients were included if they were admitted to hospital with a primary diagnosis of STEMI or NSTEMI using relevant ICD-9 and ICD-10 codes (international classification of diseases; supplementary appendix 2); STEMI and NSTEMI were identified by using validated coding algorithms with input from cardiologist authors (AB and DK).^20^ ^21^ While coding schemes were generally preserved across countries, we did allow for slight variations in accordance with local practices. The conduct and reporting of our study followed the STROBE (strengthening the reporting of observational studies in epidemiology) and RECORD (reporting of studies conducted using observational routinely collected health data) statements.^22^ ^23^

For the US, we used billing claims and enrolment files for patients enrolled in fee-for-service Medicare. For Canada, because national data are not available, we used data from Ontario and Manitoba, which encompass more than 40% of the Canadian population. For England, we used the Clinical Practice Research Datalink with Hospital Episode Statistics and death registry data from the Office for National Statistics, and for the Netherlands, we used National Basic Register Hospital Care data. For Israel, we analysed data from Clalit Health Services, which is the largest health services organisation and covers over half of the population, and for Taiwan, we used the National Health Insurance Research Database. We applied identical inclusion and exclusion criteria in the same chronological order for each country, with rare exceptions to account for local differences in data architecture.

After identifying all patients admitted to hospital with AMI, we limited our cohort to the first AMI admission during each 365 day period for each patient to avoid counting readmissions as new admissions. We excluded patients when age or sex information was missing; when their residence was outside of the jurisdiction of admission (because this would prevent evaluation of outcomes); with less than one year of data before admission or one year of follow-up data after admission (except if the reason for loss to follow-up was death); and patients with two or more months of continuous Medicare advantage enrolment during the year before or year after AMI hospital admission (US only). When patients were transferred between hospitals, we evaluated the complete episode of care. We identified comorbid conditions present on the index hospital admission and hospital admissions during the previous year using a Manitoba adaptation of the Elixhauser comorbidity index.^24^

### Outcomes

We analysed three categories of outcomes that could be reliably identified in all countries and are commonly used in the evaluation of AMI care. Firstly, we calculated the proportion of patients admitted to hospital with STEMI or NSTEMI who received cardiac intervention in hospital and within 90 days of admission; specifically, cardiac catheterisation with or without PCI including balloon angioplasty alone, PCI, and coronary artery bypass grafting (CABG). Procedures were identified using ICD-9 and ICD-10 codes, allowing some adaptation within each country (supplementary appendix 2). Secondly, we measured mortality within 30 days and one year of hospital admission. Thirdly, we evaluated measures of health system efficiency; specifically, mean and median hospital length of stay and readmission within 30 days of discharge.

### Statistical analyses

Firstly, we compared the age, sex, and selected comorbidities of patients in each jurisdiction for each calendar year; we present data from the first (2011; 2013 for Netherlands) and last year (2017) for simplicity; supplementary appendices 4-7 give full results. Because of well accepted differences in treatment approach by type of myocardial infarction, all analyses were conducted separately for the STEMI and NSTEMI patient cohorts. Secondly, we calculated STEMI and NSTEMI rates (hospital admissions per 1000 population age ≥66 years per year) for each country and calendar year, standardised to the age and sex distribution of the US AMI population in 2017 using direct standardisation.^25^ Thirdly, we compared age and sex standardised rates of cardiac interventions, mortality, mean length of stay, and readmission across countries and unadjusted median length of stay (and interquartile range).

We do not present comorbidity adjusted outcomes for several reasons. The variation in apparent rates of comorbid conditions was implausibly large. As in other studies using administrative data from different countries,^26^ ^27^ we conclude that these differences probably reflect differences in coding practices, adoption of health information technology, and payment incentives rather than true differences in the underlying prevalence of comorbid conditions in the populations of interest. More importantly, multiple studies have shown that adjustment for comorbid conditions has little overall impact on between country differences in AMI outcomes above and beyond age and sex adjustment.^28^ ^29^ We did not standardise by race and ethnicity because relevant data are not available from all countries. Moreover, race and ethnicity are social constructs that are heavily influenced by the culture and history of each country, and vary substantially across countries.^30^ Because P values can magnify the importance of clinically trivial differences, especially with the large sample sizes present in our study, and also minimise the importance of clinically important differences that do not reach a given P value threshold, we refrained from formal statistical testing.^31^ Analyses were conducted using SAS (US, Ontario, Manitoba, Taiwan), and R (Israel, England, Netherlands).

### Patient and public involvement

While patients and the public were not involved in the design, reporting, or dissemination of our study, our work was inspired by Dr Cram’s first hand experience as a practicing general internist, having worked in both the US and Canada, and the questions that patients frequently posed to him about medical care on both sides of the US-Canada border.

## Results

### Patient populations

Our full STEMI populations ranged from 5007 hospital admissions in Israel to 258 556 in the US, and NSTEMI populations ranged from 14 036 hospital admissions in Israel to 805 543 in the US (supplementary appendices 3 and 4). The rate of STEMI was lowest in England and the rates of NSTEMI were lower in England and Taiwan than all other countries throughout the study period (supplementary appendix 5). Rates of STEMI declined substantially over the study period in the US, Israel, and Taiwan, while rates of NSTEMI were generally stable across countries.

The average age of patients admitted to hospital with STEMI was approximately 78 years in 2011 but had decreased to 77 years by 2017 (table 1); patients admitted to hospital in the US and England were slightly older than patients in Canada, the Netherlands, Israel, and Taiwan (table 1). Age differences between countries were generally smaller for NSTEMI (table 1; data for all countries and years are available by request). The mean age of patients admitted to hospital for STEMI and NSTEMI decreased by approximately one year across all jurisdictions between 2011 and 2017 (table 1).

**Table 1:** Study population by condition and country for selected years

	2011							2017					
Condition and metric	US (n=161 508)	Canada (n=11 864)	England (n=8268)	Netherlands* (n=12 104)	Israel (n=2630)	Taiwan (n=7980)		US (n=146 641)	Canada (n=12 512)	England (n=6907)	Netherlands (n=14 867)	Israel (n=2783)	Taiwan (n=9450)
**STEMI**													
% of sample	20.6	25.8	20.0	46.7	29.0	48.5		22.4	27.7	29.8	33.0	24.0	35.1
Mean age (years)	78.9	77.3	78.3	76.6	78.1	77.7		77.8	76.5	77.6	76.6	76.1	76.6
Female (%)	47.5	42.9	43.1	39.9	40.6	35.8		43.1	38.8	39.7	39.3	32.5	32.4
CHF	9.1	2.5	2.1	0.2	8.9	10.5		6.7	1.8	2.9	0.1	6.5	5.6
Hypertension	76.6	41.1	55.3	5.7	59.1	22.0		80.8	46.3	58.8	7.9	52.6	16.4
Diabetes	31.2	23.3	15.8	5.0	39.6	16.2		32.5	24.5	23.9	5.9	40.2	10.6
Hypothyroidism	15.5	1.0	6.6	0.2	9.9	0.0		16.1	0.7	8.2	0.1	6.5	0.2
**NSTEMI**													
% of sample	79.4	74.2	80.0	53.3	71.0	51.1		77.6	72.3	70.2	67.0	76.0	64.9
Mean age (years)	80.3	79.6	80.3	78.0	79.9	78.3		79.5	78.9	79.8	77.7	78.8	78.2
Female (%)	50.6	46.1	45.0	41.6	46.5	42.8		47.3	42.8	41.1	39.8	40.5	41.3
CHF	17.9	7.6	7.2	0.4	17.8	17.4		17.6	6.5	9.4	0.2	14.5	15.1
Hypertension	85.2	49.3	71.2	8.1	70.0	31.6		89.7	52.3	68.4	11.4	66.0	30.1
Diabetes	40.3	33.9	28.8	7.3	50.6	25.7		43.5	37.2	33.8	9.7	57.0	20.1
Hypothyroidism	19.3	1.4	9.1	0.1	9.5	0.3		21.1	0.8	9.5	0.3	9.1	0.2

CHF=congestive heart failure; NSTEMI=non-ST elevation myocardial infarction; STEMI= ST elevation myocardial infarction.

* 2013 data shown; data unavailable for 2011-12.

Surprisingly large differences between countries were found in the proportion of AMI admissions who were women (table 1). For STEMI and NSTEMI, the proportion of female patients was 4-6% higher in the US than in all other countries and lowest in Taiwan for STEMI (32-36%) and the Netherlands for NSTEMI (39-42%). Recorded rates of comorbid conditions including congestive heart failure, hypertension, diabetes, and hypothyroidism differed considerably between countries.

### Cardiac interventions

#### STEMI

**Fig 1:**
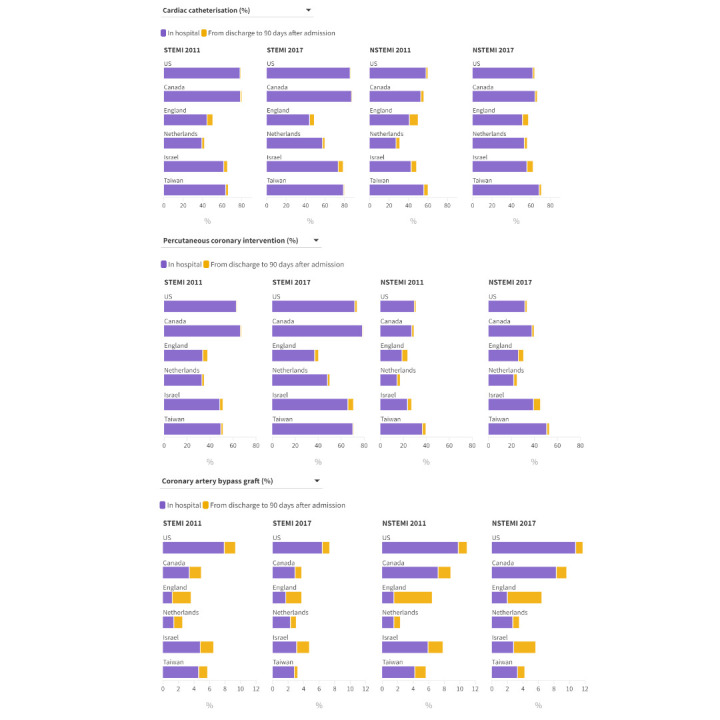
Proportion of patients receiving cardiac catheterisation, percutaneous coronary intervention, and coronary artery bypass graft surgery in hospital and within 90 days of index hospital admission. Colour coding indicates proportion of 90 day care received during index admission. Rates standardised to age and sex distribution of US acute myocardial infarction population in 2017. 2013 data shown for the Netherlands; 2011-12 data unavailable. NSTEMI=non-ST elevation myocardial infarction; STEMI=ST elevation myocardial infarction. An interactive version of this graphic is available at https://bit.ly/3vBjtMh

In 2011, use of PCI within 90 days of admission in the US and Canada (63.2% and 67.1%) was substantially higher than in all other countries (England 37.7%, Netherlands 34.6%, Israel 51.0%, Taiwan 51.1%; fig 1). By 2017, the US and Canada (73.7% and 79.0%) continued to have the highest use of PCI, while Israel (70.4%) and Taiwan (70.8%) had substantially increased their PCI use. Even in 2017, use of PCI in England and the Netherlands remained low (40.0% and 49.8%, respectively). Additional data on the use of cardiac revascularisation for all years for all countries are given in supplementary appendix 6.

The US had the highest use of CABG after STEMI throughout the study period (fig 1 and supplementary appendix 6). The use of CABG declined between 2011 and 2017 in all countries except England and the Netherlands. For instance, the use of CABG within 90 days of hospital admission in the US decreased from 9.3% in 2011 to 7.3% in 2017, and decreased from 6.5% to 4.7% in Israel, the country with the next highest use of CABG. While most CABG in the US and Canada occurred during the index hospital admission, more than half of CABG in England was performed after discharge from the initial AMI hospital admission (supplementary appendix 6).

#### NSTEMI

In 2011, use of cardiac catheterisation within 90 days of admission ranged from 30.6% in the Netherlands to 59.7% in Taiwan; in 2017, it ranged from 56.0% in the Netherlands to 70.5% in Taiwan (fig 1 and supplementary appendix 6). In 2017, use of PCI within 90 days of admission for NSTEMI differed more than twofold between countries, with the lowest use in the Netherlands (24.7%) and the highest in Taiwan (52.7%).

In 2011, CABG within 90 days of admission was highest in the US and Canada (10.8% and 8.8%). By 2017, CABG had increased to 11.7% in the US and 9.6% in Canada, but it was only 6.4% in England, which was the third highest country. Between 2011 and 2017, CABG use after NSTEMI increased by a clinically meaningful amount in three countries, decreased in two, and remained stable in one.

### Mortality

**Fig 2:**
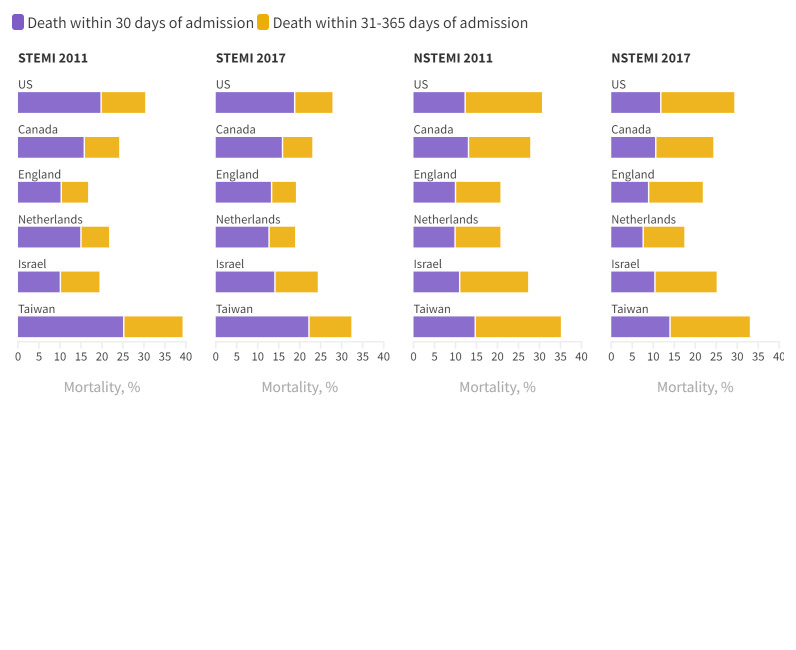
Mortality within 30 days and one year of index hospital admission for acute myocardial infarction. Colour coding indicates proportion of deaths occurring within 30 days and 31-365 days of admission. Rates standardised to age and sex distribution of US acute myocardial infarction population in 2017. 2013 data shown for the Netherlands; 2011-12 data unavailable. NSTEMI=non-ST elevation myocardial infarction; STEMI=ST elevation myocardial infarction. An interactive version of this graphic is available at https://bit.ly/3v2W1bO

Mortality after STEMI and NSTEMI was higher in the US and Taiwan than the other four countries in 2011 and 2017 at 30 days and one year (fig 2, supplementary appendix 7). For example, in 2017, 30 day STEMI mortality was 18.8% in the US, 22.2% in Taiwan, and 15.9% in Canada. Similarly in 2017, one year NSTEMI mortality was 29.3% in the US, 33.0% in Taiwan, and 25.1% in Israel (fig 2). Between 2011 and 2017, one year mortality after STEMI and NSTEMI decreased by 1% or more in four countries.

### Hospital efficiency

**Fig 3:**
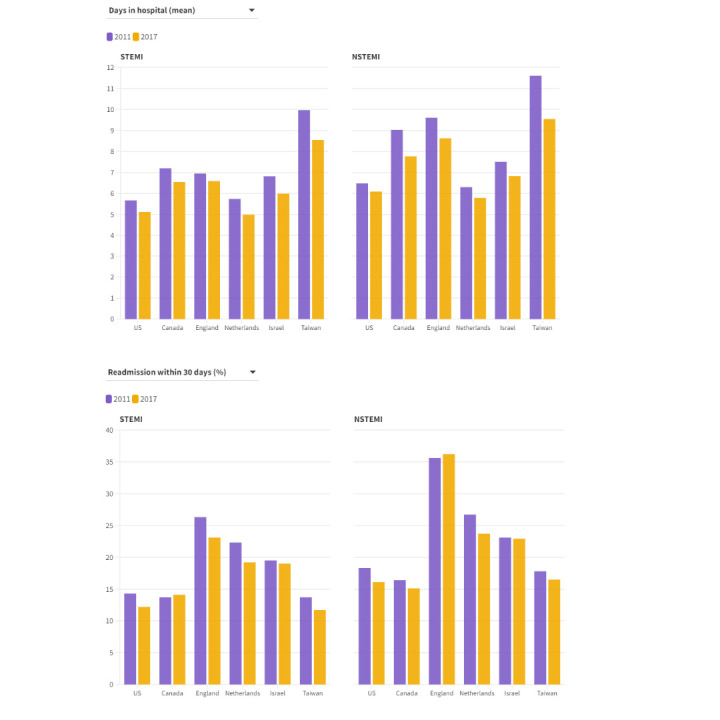
Measures of health system efficiency. Hospital length of stay and 30 day readmission rate for STEMI and NSTEMI by country. Rates standardised to age and sex distribution of US acute myocardial infarction population in 2017. 2013 data shown for the Netherlands; 2011-12 data unavailable. NSTEMI=non-ST elevation myocardial infarction; STEMI=ST elevation myocardial infarction. An interactive version of this graphic is available at https://bit.ly/3OGAFJd

Marked variation was found among the countries for mean length of stay and 30 day readmission rate for STEMI and NSTEMI (fig 3, supplementary appendices 6 and 7). For example, in 2017, mean length of stay for STEMI ranged from 5.0 days (Netherlands) and 5.1 days (US) to 8.5 days (Taiwan), while median length of stay ranged from 3 to 6 days across countries (supplementary appendix 6). The 2017 30 day readmission rate for STEMI ranged from 11.7% (Taiwan) and 12.2% (US) to 23.1% in England. The US was unique among countries in having short hospital length of stay and low 30 day readmission rates for STEMI and NSTEMI. Between 2011 and 2017, hospital length of stay decreased for STEMI and NSTEMI by approximately one day in all countries; 30 day readmission rates decreased substantially for STEMI and NSTEMI in the US, the Netherlands, and Taiwan but less in the other countries.

## Discussion

### Principal findings

In an analysis of patient level nationally representative administrative data from six high income countries, we observed marked differences between countries in the management of AMI and patient outcomes. We found substantial differences in the use of revascularisation, suggestive of potential underuse of PCI for STEMI in England and potential overuse of CABG in the US. Our finding of substantially higher mortality in the US and Taiwan for STEMI and NSTEMI compared with the other countries is concerning. With respect to efficiency, the US achieved comparatively short hospital length of stay and low 30 day hospital readmission rates. All countries had areas where performance was relatively better and areas where performance was relatively worse. No country excelled in all performance indicators. In aggregate, our findings provide the type of empirical data that should guide countries in focusing their improvement efforts.

Several findings warrant discussion. Firstly, there were large between country differences in the use of PCI and CABG, which is noteworthy. The benefits of early PCI in patients with STEMI have been shown repeatedly for nearly two decades; these benefits are reflected in international practice guidelines.^32^ ^33^ ^34^ ^35^ Therefore, our finding that in 2017 65-80% of patients admitted to hospital for STEMI in the US, Israel, and Canada received PCI in hospital compared with 35% in England and 50% in the Netherlands deserves further consideration. The low use of PCI in England and the Netherlands is striking and expands upon a small number of previous studies suggesting potentially low use in these countries.^15^ ^36^ ^37^ The differences between countries are probably a manifestation of those that have prioritised the development, funding, maintenance, and monitoring of primary PCI programmes (US, Canada, and Israel) and countries that have not (England and the Netherlands).^15^ ^36^ ^37^ ^38^ ^39^ ^40^ ^41^

Our finding that CABG use in the US was 50-100% higher than in other countries also raises questions. Current national and international guidelines recommend CABG in a narrow subset of patients, such as patients with diabetes and extensive coronary artery disease, or those with coronary anatomy that is not amenable to PCI.^42^ ^43^ While it is possible that higher CABG use in the US reflects greater patient clinical complexity or acuity (eg, cardiogenic shock, severe heart failure, multivessel disease), a more plausible explanation is that the unique combination of a permissive regulatory environment in the US in combination with high Medicare reimbursement results in greater use of CABG in the US.^44^ Alternatively, the low use of CABG in England and the Netherlands could reflect underuse and decreased availability of CABG surgery.^45^ Given the implications of potential overuse of CABG in the US (surgical morbidity and mortality, cost) or underuse in England and the Netherlands, confirmation of our findings using clinical registries or enhanced administrative data would be important.

Secondly, our findings relating to mortality require careful consideration. For the STEMI and NSTEMI cohorts, age and sex standardised mortality within one year of hospital admission was 5-10% (absolute difference) higher in the US and Taiwan than in the other countries. The higher mortality in the US and Taiwan cannot be explained by lower use of revascularisation, as shown in our study. While differential rates of smoking are an attractive explanation for increased mortality in the US and Taiwan, data from the Global Burden of Disease Study group and OECD suggest relatively modest smoking rates in the US compared with the other IHSRC countries, though smoking rates in Taiwan are fairly high, particularly among men.^46^ ^47^ While large international comparative studies of secondary prevention strategies are limited, data are not clear and convincing that use of statins, angiotensin converting enzyme inhibitors, and β blockers are markedly lower in the US and Taiwan^48^ ^49^ ^50^ ^51^ than countries with lower mortality.^8^ ^52^ While it is possible that poorer outcomes in the US and Taiwan can be attributable to social determinants of health, such a supposition requires rigorous evaluation.

Thirdly, our findings of substantial differences in measures of efficiency are important. Over the past decade the US has devoted substantial resources towards reducing hospital readmissions under the assumption that readmission rates in the US are too high and signal low quality care; interestingly, few direct cross country comparisons exist that support this assumption.^53^ Our finding that hospital readmission rates in the US were substantially lower than in other countries challenges the assumption that further reductions in US readmission rates are feasible or advisable. Though Taiwan and Canada had readmission rates that were close to those in the US, these countries had substantially longer length of stay; longer length of stay in Canada and England has been recognised as a chronic problem and might relate to a shortage of post-acute-care beds.^54^ ^55^ Therefore, from an efficiency standpoint, the US was able to achieve a relatively low 30 day readmission rate while simultaneously maintaining a lower hospital length of stay than other countries. Taken together, these findings suggest that hospital throughput in the US is relatively efficient. Alternatively, England, with long hospital length of stay and high readmission rates, would seem to have opportunities for improved efficiency. In evaluating efficiency, it is important to mention our decision not to focus on monetary costs.^56^ ^57^ ^58^ The countries included in the IHSRC use different models of physician payment (eg, salaried *v* fee for service), hospital payment (eg, episode based *v* global budgeting), and input costs (drugs and devices).^59^ ^60^ ^61^ Given these differences, we chose to compare use of costly AMI interventions (eg, PCI, CABG) and measures of efficiency (readmission, length of stay) that are not subject to the differences in how countries assign and measure costs.

Fourthly, our finding that the percentage of female patients with STEMI and NSTEMI in the US was 4-6% higher than in all other countries warrants attention. This finding could reflect a higher prevalence of cardiovascular risk factors (obesity, diabetes, smoking) in US women relative to their international peers.^62^ Another possibility would be overdiagnosis of AMI in US women or underdiagnosis of AMI in women in other countries. All possibilities warrant investigation. Finally, Israel achieved comparable if not better performance than other countries for revascularisation, mortality, and efficiency, despite far lower spending.^63^

### Policy implications

Our analysis has important implications for international health system comparisons.^1^ Several recent papers and reports have provided high level comparisons of health system performance across high income countries.^3^ ^4^ ^64^ However, these analyses rely on aggregated population level data and lack the granularity required to evaluate disease specific care processes or outcomes.^13^ ^18^ Several European collaboratives including the Echo consortium and Eurohope have compared AMI care across countries, but did not differentiate STEMI from NSTEMI and did not include data from the US or Canada.^15^ ^17^ Recently the ICCONIC collaborative has published several rigorous international comparisons primarily focused around costs of care for patients admitted to hospital with hip fracture and multimorbidity.^18^ ^57^ ^65^ ^66^

### Strengths and limitations of our study

Our work adds to the existing international comparison research landscape. We developed the IHSRC using analytic teams with extensive local expertise located in each country who analyse their own patient level data while closely following a common protocol for identifying patients and all variables of interest. Strengths of this approach include our ability to identify similar patient cohorts in each country and establishing rigorous methods for assessing outcomes using standardised definitions. Despite the numerous strengths of this approach, we encountered certain challenges. For example, changes in data formatting and availability within the Netherlands forced us to exclude patients admitted to hospital in 2011 and 2012, and the US switched from ICD-9 to ICD-10 diagnosis codes in late 2015. Additionally, we found large differences in the rates of comorbid illness irrespective of the coding algorithms and methods used; these differences probably reflect fundamental differences between countries in the attention and resources devoted to coding and documentation.

Our study has several additional limitations. Firstly, we relied on administrative data and lacked detailed clinical information about myocardial infarction severity or treatments. While we meticulously followed well established coding algorithms for identification of AMI and all outcomes after myocardial infarction, not all codes have been validated and countries were encouraged to adapt our general coding scheme to local practice. Secondly, we did not adjust for race or ethnicity. Race and ethnicity data were not available for all countries; moreover, race and ethnicity are increasingly recognised as a social construct and it would be difficult (and potentially wrong) to attempt to equate findings related to racial and ethnic minority groups in one country (eg, Taiwan) with people of the same minority group living in another country (eg, the US or the Netherlands). Thirdly, although we used patient level administrative data from each country, we lacked certain types of data from some countries (eg, pharmacy data, level of independence before hospital admission, discharge destination) and so we could not assess certain important aspects of AMI care. Similarly, AMI is a single condition and comprehensive between country comparisons should ultimately span many additional conditions and domains that we were not able to examine in the current analysis, including patient experience and patient reported outcomes, access, and equity.^67^ ^68^ Fourthly, our study was limited to adults aged 66 years and older who were admitted to hospital for AMI and so the findings might not apply to younger patients or those with private insurance or enrolled in Medicare managed care in the US. Similarly, because our study focused on patients admitted to hospital with a primary diagnosis of AMI, our per capita rates of AMI could be influenced if countries systematically differed in the proportion of patients with AMI presenting to hospital or those who were misclassified or misdiagnosed.

### Conclusions

In our analysis of nationally representative patient level data for people admitted to hospital with AMI in six high income countries, we found clinically important differences between countries in three domains of care as assessed by rates of cardiac interventions, mortality, hospital length of stay, and 30 day readmission rates. While all countries had areas of high performance, no country excelled in all three domains. Therefore, our results suggest that all countries have important opportunities for improvement.

**What is already known on this topic**

Aggregated population level data have shown clinically important differences in life expectancy and healthcare spending across high income countries
High income countries have different approaches to delivering healthcare to their populations
Very few studies have evaluated differences between countries in treatments and outcomes for patients admitted to hospital with a common medical condition

**What this study adds**

For patients admitted to hospital with acute myocardial infarction in six high income countries, substantial differences were found between countries in use of cardiac revascularisation, mortality, hospital length of stay, and hospital readmissions
Findings include low use of revascularisation for ST elevation myocardial infarction in England and the Netherlands, and high one year mortality rates in the United States and Taiwan
Considerable differences were found in care of patients with acute myocardial infarction in high income countries with well developed healthcare systems

## Data Availability

Many details of the study protocol and coding schemes are available in appendices 1 and 2. Datasets are typically unavailable for sharing because of privacy policies and regulations of participant countries. Additional data, analysis coding, and aspects of our protocol might be available by request from the corresponding author, depending upon the regulatory environment of IHSRC participant countries.

## Web extra

Extra material supplied by authors

Web appendix: Supplementary appendices

## Notes

Contributors: PC and BL acted as principal investigators; they take responsibility for the integrity of the data and the accuracy of the data analysis. PC, BL, LAH, DK, LML, VN, TAS, CUdG were responsible for the study concept and design, and supervised the study. All authors contributed to the acquisition, analysis, or interpretation of the data; and critical revision of the manuscript for important intellectual content. PC and BL drafted the manuscript. LAH, PB, CF, MG, RH, NH, LP, FQ, and LY conducted the statistical analysis. All authors provided administrative, technical, and material support. PC and BL are the guarantors. The corresponding author attests that all listed authors meet authorship criteria and that no others meeting the criteria have been omitted.

The lead authors (PC and BL) affirm that the manuscript is an honest, accurate, and transparent account of the study being reported; that no important aspects of the study have been omitted; and that any discrepancies from the study as planned have been explained.

Dissemination to participants and related patient and public communities: These results will not be directly shared with the patients identified in this retrospective analysis. Our results will be shared with our primary funding agency (US National Institute on Aging) and results will also be disseminated by our investigators in their respective countries through their home institutions.

Provenance and peer review: Not commissioned; externally peer reviewed.
