# Supplementary material for: Variation in revascularisation use and outcomes of patients in hospital with acute myocardial infarction across six high income countries: cross sectional cohort study
Source: BMJ. 2022 May 4;377:e069164. doi: 10.1136/bmj-2021-069164 (PMC9066381; doi:10.1136/bmj-2021-069164)
Supplement: Supplementary file 1 — Web appendix: Supplementary appendices [file crpe069164.ww.pdf]

Appendix 1: Description of data sources and research ethics approval from each country/jurisdiction.

United States

All hospitalizations for AMI in adults age > 66 years at the time of hospitalization during calendar years 2011-2017 were identified using 100% Medicare fee-for-service (FFS) data.[1] Data from 2010 was used as a "look-back" and data from 2018 were used to ascertain post-AMI outcomes.

During our study period approximately between 75% (2011) and 67% (2017) of the US Medicare population were enrolled in FFS Medicare during this period and thus included in our study population with the remainder enrolled in Medicare managed care plans.[2]

**AMI Identification and Outcomes**	
Data sources[1]	Use
100% Medicare Part A Data (2010-2018)	a. Identification of patients hospitalized with a primary diagnosis of AMI[3-5]<br>b. Creation of comorbidities<br>c. In-hospital and post-hospital utilization and outcomes
100% Medicare Beneficiary Summary File	a. Date of death
100% Medicare FFS Inpatient Data	PCI on the admission day<br>(Note that procedure date is available in Inpatient data but not in Medpar)
**Population Count and Demographics**<br>(Used for Calculation of AMI Hospitalization Rates and Standardization)	
Medicare Beneficiary Summary File	Number of Medicare FFS enrollees by year<br>Age/race/sex/ethnicity/area of residence information

Analyses were conducted at Harvard Medical School. This study was approved by the Institutional Review Board (IRB) of the Harvard Faculty of Medicine.

[1] For more information on US Medicare Part A Data please visit: https://www.cms.gov/Research-Statistics-Data-and-Systems/Statistics-Trends-and-Reports/MedicareFeeforSvcPartsAB/MEDPAR

Ontario (Canada)

All hospitalizations for AMI in adults age > 66 years at the time of hospitalization during calendar years 2011-2017 were identified using the Discharge Abstract Database (DAD) for Ontario. Data from 2010 were used as a "look-back" and data from 2018 were used to ascertain post-AMI outcomes.

The DAD includes all admissions to all acute care hospitals in the province of Ontario and thus is inclusive of the entire Ontario population.

Datasets used in the analyses were linked using unique encoded identifiers and analyzed at ICES. ICES is an independent, non-profit research institute whose legal status under Ontario's health information privacy law allows it to collect and analyze health care and demographic data, without consent, for health system evaluation and improvement.[6]

**AMI Identification and Outcomes**	
Data source[2]	Use
Discharge Abstract Database (Ontario), Canadian Institute for Health Information (2010-2018)	a. Identification of patients hospitalized with a primary diagnosis of AMI [7 8]<br>b. Creation of comorbidities<br>c. In-hospital outcomes
OHIP Billing Data (2011-2018)	a. Post-AMI treatments and procedures
Registered Persons Data Base files	a. Determination of death date, birth date, and insurance coverage start and end date
**Population Count and Demographics** (Used for Calculation of AMI Hospitalization Rates and Standardization)	
Registered Persons Data Base files	Age/sex/ethnicity/area of residence information

The use of the data in this project is authorized under section 45 of Ontario's Personal Health Information Protection Act (PHIPA) and does not require review by a Research Ethics Board.

The analyses, conclusions, opinions and statements expressed herein are solely those of the authors and do not reflect those of the funding or data sources; no endorsement is intended or should be inferred.

[2] For more information on Ontario Discharge Abstract Data please visit https://data.ontario.ca/dataset/discharge-abstract-database-dad-ontario-hospitals

Manitoba (Canada)

All hospitalizations for AMI in adults age > 66 years at the time of hospitalization during calendar years 2011-2017 were identified using 100% Manitoba Discharge Abstract Database (DAD). Data from 2010 was used as a "look-back" and data from 2018 were used to ascertain post-AMI outcomes.

The Manitoba DAD includes all admissions to all acute care hospitals in the province of Manitoba and thus is inclusive of the entire Manitoba population eligible to receive health services.

Analyses were conducted using the Population Research Data Repository housed at the Manitoba Centre for Health Policy and utilized the administrative data from the Manitoba ministry of health (I.e., Manitoba Health and Seniors Care). The administrative data captures all publicly-insured health services for all residents of the province who are eligible to receive health services.

**AMI Identification and Outcomes**	
Data source[3]	Use
Manitoba Discharge Abstract Database (2010-2018)	a. Identification of patients hospitalized with a primary diagnosis of AMI[8]<br>b. Creation of comorbidities<br>c. In-hospital outcomes
Physician Billing Data (2011-2018)	a. Post-AMI treatments and procedures
Manitoba Health Insurance Registry	Determination of death date, birth date, and insurance coverage start and end dates.
**Population Count and Demographics**<br>(Used for Calculation of AMI Hospitalization Rates and Standardization)	
Manitoba Health Population Database	Counts of population by sex, age group, and year

The authors acknowledge the Manitoba Centre for Health Policy for use of data contained in the Manitoba Population Health Research Data Repository (Health Information Privacy Committee project # 2019/2020-38). The results and conclusions are those of the authors and no official endorsement by the Manitoba Centre for Health Policy, Manitoba Health and Seniors Care, or other data providers is intended or should be inferred.

[3] For more information on Manitoba data please visit: https://umanitoba.ca/manitoba-centre-for-health-policy/data-repository

England

All hospitalizations for AMI in adults age > 66 years at the time of hospitalization during calendar years 2011-2017 were identified using the Clinical Practice Research Datalink. Data from 2010 was used as a "look-back" and data from 2018 were used to ascertain post-AMI outcomes. CPRD has been shown to be representative of the English population by age, sex and ethnicity, and validated for research.[9-11] Analyses were conducted at the Institute of Health Informatics, University College London using linked electronic health records from primary care (CPRD), hospitalisation (HES) and the national death registry (ONS).[10]

**AMI Identification and Outcomes**	
Data source	Use
Clinical Practice Research Datalink (GOLD and Aurum)	a. Patient identification[9]<br>b. Demographics<br>c. Creation of comorbidities
Hospital Episode Statistics (HES)	a. Identification of patients hospitalized with a primary diagnosis of AMI[9 12]<br>b. Creation of comorbidities<br>c. In-hospital outcomes<br>d. Inpatient procedures
Office of National Statistics	Cause-specific mortality
**Population Count and Demographics**<br>(Used for Calculation of AMI Hospitalization Rates and Standardization)	
	Counts of population by sex, age group, and year

The study was approved by the MHRA (UK) Independent Scientific Advisory Committee 20_021, under Section 251 (NHS Social Care Act 2006). This study is based in part on data from the Clinical Practice Research Datalink obtained under licence from the UK Medicines and Healthcare products Regulatory Agency. The data is provided by patients and collected by the NHS as part of their care and support. The interpretation and conclusions contained in this study are those of the author/s alone. HES and ONS data copyright 2022, re-used with the permission of The Health & Social Care Information Centre. All rights reserved.

Netherlands

All hospitalizations for AMI in Dutch adults age > 66 years at the time of hospitalization during calendar years 2013-2017 were identified using data from the national register for hospital care.[13] Data from 2012 was used as a "look-back" and data from 2018 were used to ascertain post-AMI outcomes.

These data include all inpatient hospitalizations for the Netherlands. Primary and secondary diagnosis are recorded as well as the main procedure performed during the admission. Demographic information and, if applicable, date of death, were extracted from municipality registers[4].

**AMI Identification and Outcomes**	
Data sources[5]	Use
National register for hospital care, Landelijke Basisregistratie Ziekenhuiszorg (LBZ – 2013-2017) and Landelijke Medische Registratie (LMR – 2012)	a. Identification of patients hospitalized with a primary diagnosis of AMI<br>b. Creation of comorbidities<br>c. In-hospital and post-hospital procedure utilization[6] and outcomes
Municipality Register (GBAPERSOONTAB, GBAOVERLIJDENTAB)	Determination of age and sex, date of death
**Population Count and Demographics**<br>(Used for Calculation of AMI Hospitalization Rates and Standardization)	
Municipality Register (GBA)	Counts of population by sex, age group, and year

This study was approved by the Internal Review Board (IRB) of the Erasmus School of Health Policy and Management on October 5, 2019.

[4] An example of another paper using the same data is García-Gómez, P., van Kippersluis, H., O'Donnell, O., & van Doorslaer, E. (2013). Long Term and Spillover Effects of Health Shocks on Employment and Income. *The Journal of human resources*, *48*(4), 873–909. https://doi.org/10.1353/jhr.2013.0031

[5] Statistics Netherlands (2022) provides detailed information on the variables and observations in these data sources, their representativity, and the procedure to get data access.

[6] The procedures were classified using the CBV, CVV and ZA classifications. Procedure codes occurring in the cohort were translated with the "verrichtingenthesaurus" (https://trex.dhd.nl/). Based on the translation these three classifications were assigned. Similar approach was followed by Rabbe et al. (in press).

Israel

Analyses were conducted at the Clinical Research Center in Soroka University Medical Center and included national data of Clalit Health Services insured patients.

All hospitalizations for AMI in adults age > 66 years at the time of hospitalization during calendar years 2011-2017 were identified using Clalit Health Services (CHS) Data sharing platform powered by MDClone © (https://www.mdclone.com).[16] Data from 2010 was used as a "look-back" and data from 2018 were used to ascertain post-AMI outcomes. The data is broadly representative of the Israel population with respect to age, sex, and geography. Clalit Health Services is Israel's largest insurance company and health care provider, providing most of Israel's health care services and providing health insurance to 54% of the country's population.[14] Services include primary, secondary, and tertiary care (including a third of Israel's acute care beds), as well as pharmacies and paramedical services. CHS maintains a comprehensive database, continuously updated with information about a subject's demographics, community and outpatient visits, laboratory tests, hospitalizations, medication prescriptions, and purchases.

**AMI Identification and Outcomes**	
Data sources[7]	Use
CHS Data warehouse using accessed by MDClone©	a. Identification of patients hospitalized with a primary diagnosis of AMI<br>b. Demographic information (Age, sex, SES)<br>c. Creation of comorbidities
CHS Data warehouse using accessed by MDClone©	d. procedures related to the diagnosis of AMI<br>e. In-hospital and post-hospital utilization and outcomes
**Population Count and Demographics** (Used for Calculation of AMI Hospitalization Rates and Standardization)	
CHS Data warehouse using accessed by MDClone©	Population denominator includes all Clalit members that were > 66 years in the period of 2011-2017 and were members in Clalit one year before and after this period.

This study was conducted according to the guidelines of the Declaration of Helsinki was approved by the Institutional Review Board of Soroka University Medical Center (Ref. 0467-18)

[7] For more information on the Israel data from Clalit please see: http://clalitresearch.org/about-us/our-data/ Accessed February 22, 2022

Taiwan:

All hospitalizations for AMI in adults age > 66 years at the time of hospitalization during calendar years 2011-2017 were identified using Taiwan's Inpatient Expenditures data. Data from 2010 was used as a "look-back" and data from 2018 were used to ascertain post-AMI outcomes.

Key data files included the National Health Insurance (NHI) Inpatient Expenditures by Admission file, which contains all admissions to all acute care hospitals in Taiwan and thus is inclusive of Taiwan population. The NHI Registry for Beneficiaries file includes demographic and socioeconomic status information of all NHI beneficiaries. Because all legal residents in Taiwan are eligible to enroll in the NHI program and the enrollment rate has exceeded 99% since 2000, these data are population representative. The Cause of Death Data provides information on cause and date of all reported deaths in Taiwan. Analyses were conducted at the Yang-Ming branch of the Health and Welfare Data Science Center, Taiwan Ministry of Health and Welfare. The main data sources were the National Health Insurance Research Database and Cause of Death Data.[15]

**AMI Identification and Outcomes**	
Data sources[8]	Use
Inpatient Expenditures by Admissions (2010-2018)	a. Identification of patients hospitalized with a primary diagnosis of AMI[16 17]<br>b. Creation of comorbidities<br>c. In-hospital and post-hospital utilization and outcomes
Cause of Death Data	Determination of death date
**Population Count and Demographics**<br>(Used for Calculation of AMI Hospitalization Rates and Standardization)	
Registry for Beneficiaries	a. Counts of population by sex, age group, and year<br>b. Determination of birth date and sex

This study was approved by the Institutional Review Board of National Yang Ming Chiao Tung University (IRB number: YM110134E).

[8] For more information on US Medicare Part A DataTaiwan's NHI Data and Cause of Death Data please visit: https://dep.mohw.gov.tw/dos/cp-5119-59201-113.html (in Chinese)

References

1. Rosenkrantz AB, Hughes DR, Duszak R, Jr. Medicare Claims Data Resources: A Primer for Policy-Focused Radiology Health Services Researchers. *J Am Coll Radiol* 2017;14(12):1538-44. doi: 10.1016/j.jacr.2017.04.005 [published Online First: 2017/06/02]
2. Neuman P, Jacobson GA. Medicare Advantage Checkup. *New England Journal of Medicine* 2018;379(22):2163-72. doi: 10.1056/NEJMhpr1804089
3. Likosky DS, Zhou W, Malenka DJ, et al. Growth in medicare expenditures for patients with acute myocardial infarction: a comparison of 1998 through 1999 and 2008. *JAMA Intern Med* 2013;173(22):2055-61. doi: 10.1001/jamainternmed.2013.10789 [published Online First: 2013/09/26]
4. Popescu I, Nallamothu BK, Vaughan-Sarrazin MS, et al. Racial differences in admissions to high-quality hospitals for coronary heart disease. *Arch Intern Med* 2010;170(14):1209-15. doi: 10.1001/archinternmed.2010.227 [published Online First: 2010/07/28]
5. Levy AE, Hammes A, Anoff DL, et al. Acute Myocardial Infarction Cohorts Defined by International Classification of Diseases, Tenth Revision Versus Diagnosis-Related Groups: Analysis of Diagnostic Agreement and Quality Measures in an Integrated Health System. *Circ Cardiovasc Qual Outcomes* 2021;14(3):e006570. doi: 10.1161/circoutcomes.120.006570 [published Online First: 2021/03/04]
6. Ghali WA, Rothwell DM, Quan H, et al. A Canadian comparison of data sources for coronary artery bypass surgery outcome "report cards". *Am Heart J* 2000;140(3):402-8. doi: 10.1067/mhj.2000.109222 [published Online First: 2000/08/31]
7. Ko DT, Khera R, Lau G, et al. Readmission and Mortality After Hospitalization for Myocardial Infarction and Heart Failure. *J Am Coll Cardiol* 2020;75(7):736-46. doi: 10.1016/j.jacc.2019.12.026 [published Online First: 2020/02/23]
8. Ko DT, Ahmed T, Austin PC, et al. Development of Acute Myocardial Infarction Mortality and Readmission Models for Public Reporting on Hospital Performance in Canada. *CJC Open* 2021;3(8):1051-59. doi: 10.1016/j.cjco.2021.04.012 [published Online First: 2021/09/11]
9. Herrett E, Shah AD, Boggon R, et al. Completeness and diagnostic validity of recording acute myocardial infarction events in primary care, hospital care, disease registry, and national mortality records: cohort study. *Bmj* 2013;346:f2350. doi: 10.1136/bmj.f2350 [published Online First: 2013/05/23]
10. Herrett E, Gallagher AM, Bhaskaran K, et al. Data Resource Profile: Clinical Practice Research Datalink (CPRD). *Int J Epidemiol* 2015;44(3):827-36. doi: 10.1093/ije/dyv098 [published Online First: 2015/06/08]
11. Herrett E, Gallagher AM, Bhaskaran K, et al. Data Resource Profile: Clinical Practice Research Datalink (CPRD). *International Journal of Epidemiology* 2015;44(3):827-36. doi: 10.1093/ije/dyv098
12. Nedkoff L, Lopez D, Goldacre M, et al. Identification of myocardial infarction type from electronic hospital data in England and Australia: a comparative data linkage study. *BMJ Open* 2017;7(11):e019217. doi: 10.1136/bmjopen-2017-019217
13. World Health Organization. Regional Office for E, European Observatory on Health S, Policies, et al. Netherlands: health system review. Copenhagen: World Health Organization. Regional Office for Europe 2016:240 p.

14. Benis A, Harel N, Barak Barkan R, et al. Patterns of Patients' Interactions With a Health Care Organization and Their Impacts on Health Quality Measurements: Protocol for a Retrospective Cohort Study. *JMIR Res Protoc* 2018;7(11):e10734. doi: 10.2196/10734 [published Online First: 2018/11/09]
15. Hsieh CY, Su CC, Shao SC, et al. Taiwan's National Health Insurance Research Database: past and future. *Clin Epidemiol* 2019;11:349-58. doi: 10.2147/clep.S196293 [published Online First: 2019/05/24]
16. Lee CH, Fang CC, Tsai LM, et al. Patterns of Acute Myocardial Infarction in Taiwan from 2009 to 2015. *Am J Cardiol* 2018;122(12):1996-2004. doi: 10.1016/j.amjcard.2018.08.047 [published Online First: 2018/10/12]
17. Saw SM, Hong CY, Lee J, et al. Awareness and health beliefs of women towards osteoporosis. *Osteoporos Int* 2003;14(7):595-601.

Appendix 2: List of codes used by each country to identify STEMI, NSTEMI, PCI, diagnostic catheterization (cardiac catheterization without intervention), and CABG[9]

	United States	Canada	England	Netherlands	Israel	Taiwan
STEMI	Icd-9:<br>41000<br>41001<br>41010<br>41011<br>41020<br>41021<br>41030<br>41031<br>41040<br>41041<br>41050<br>41051<br>41060<br>41061<br>41080<br>41081<br>41090<br>41091<br><br>Icd-10:<br>I2101<br>I2102<br>I2109<br>I2111<br>I2119<br>I2121<br>I2129<br>I213<br>I219	ICD-10:<br>Main diagnosis I21, I22 and with secondary R9430	ICD-10:<br>I210, I211, I212, I213	Icd-9:<br>410<br>4100<br>4102<br>4103<br>4104<br>4105<br>4106<br><br>Icd-10:<br>I21<br>I210<br>I211<br>I212<br>I213	ICD-9:<br>41000<br>41001<br>41010<br>41020<br>41021<br>41030<br>41031<br>41040<br>41041<br>41050<br>41051<br>41060<br>41061	ICD-9:<br>41000<br>41001<br>41010<br>41011<br>41020<br>41021<br>41030<br>41031<br>41040<br>41041<br>41050<br>41051<br>41060<br>41061<br>41080<br>41081<br>41090<br>41091<br><br>ICD-10:<br>I2101<br>I2102<br>I2109<br>I2111<br>I2119<br>I2121<br>I2129<br>I213<br>I219
NSTEMI	Icd-9:<br>41070<br>41071	ICD-10:<br>Main diagnosis I21, I22 with exclude secondary R9430	ICD-10:<br><br>I214, I219, I220, I221, I228, I229	Icd-9:<br>4107<br>4108<br>4109	ICD9:<br>41002<br>41012<br>41022	ICD-9:<br>41070<br>41071

[9] Within a general framework countries were allowed to adapt coding schemes to fit local context and practice

	Icd-10:<br>I214<br>I21A1<br>I21A9			Icd-10<br>I214<br>I2140<br>I2141<br>I2142<br>I2149<br>I220<br>I221<br>I228<br>I229<br>I219	41032<br>41042<br>41052<br>41062<br>4107<br>41070<br>41071<br>41072<br>41080<br>41081<br>41082<br>41090<br>41091<br>41092	Icd-10:<br>I214<br>I220<br>I221<br>I228<br>I229
PCI	Icd-9:<br>0.66, 36.06, 36.07, 36.09<br>ICD-10:<br>027x3	CCI:<br>1IJ50, 1IJ54,<br>1IJ57GQ	OPCS-4:<br>K47.1, K49, K49.1, K49.2, K49.3, K49.4, K49.8, K49.9, K50, K50.1, K50.4, K50.8, K50.9, K75, K75.1, K75.2, K75.3, K75.4, K75.8, K75.9	ZA codes:<br>033232<br>033231<br>033238<br>080827<br>033233<br>033234<br>033235<br><br>CVV codes:<br>88370<br>88374<br>88371<br>88372<br>88375<br>88376<br>88377<br>88378<br>88379<br>8839<br>8837<br><br>CBV codes:<br>333107D<br>333108A<br>333108C<br>333108K	Z3606<br>Z36060<br>Z3607<br>Z36070	Icd-9:<br>36.06, 36.07<br><br>ICD-10:<br>027x3

				333109S<br>333109T<br>333208<br>333234I<br>333299C<br>333107A<br>333107B<br>333107C<br>333107G<br>333108B<br>333108I<br>333109A<br>333109B<br>333109I<br>333109K<br>333109O<br>333109Q<br>333297<br>333297B<br>333297C<br>333297D<br>333297E<br>333297I<br>333602T<br>380029B<br>381029B<br>381729W<br>381729Z<br>333108G<br>333109U<br>333109V<br>333109W<br>333109X<br>333108J<br>333108H<br>333043B		
Diagnostic catheterization	Icd-9:,37.21, 37.22, 37.23, Icd-10:4A02	CCI:<br>3IP10 (with accompanying billing code Z442, G297)	OPCS-4:<br>K65, K631, K632, K633, K634, K635, K636	ZA codes:<br>033229<br>033219<br><br>CVV codes:	Z3721<br>Z37211<br>Z3722<br>Z3723	Icd-9:<br>37.21, 37.22, 37.23,<br>Icd-10:<br>4A02

				12750<br>12751<br>12758<br>12759<br>1273<br>1274<br>1275<br>1276<br>12760<br>12761<br>1277<br>1278<br><br>CBV codes:<br>333203A<br>333204<br>333205<br>333206<br>333207C<br>333207D<br>333207E<br>333207F<br>333207G<br>333207J<br>333222<br>333207B<br>333201B<br>333212<br>333291B<br>333291<br>333291<br>333201A<br>333200<br>333209<br>333210S<br>339845E		
CABG	Icd-9:<br>36.1x, Icd-10: 021x	CCI:<br>1IJ76	OPCS-4:<br>K40, K40.1, K40.2, K40.3, K40.4, K40.8, K40.9, K41, K41.1, K41.2, K41.3, K41.4,	ZA codes:<br>033105<br>033100<br>033101<br>033102	Z3610<br>Z3613<br>Z3614<br>Z3615<br>Z3616	Icd-9: 36.1x<br><br>Icd-10:<br>021x

			K41.8, K41.9, K42, K42.1, K42.2, K42.3, K42.4, K42.8, K42.9, K43, K43.1, K43.2, K43.3, K43.4, K43.8, K43.9, K44, K44.1, K44.8, K44.9, K45, K45.1, K45.2, K45.3, K45.4, K45.5, K45.8, K45.9, K46, K46.1, K46.2, K46.3, K46.4, K46.8, K46.9, K44.2, K45.6, K46.5	033103<br>033104<br>033106<br>033107<br><br>CVV codes:<br>5361<br>53610<br>53611<br>53619<br>53618<br>53612<br>536120<br>536121<br>536122<br><br>CBV codes:<br>333102<br>333100<br>333105A<br>333103<br>333102A<br>333184A<br>333184H<br>333553<br>333104A<br>333105H<br>333105C<br>333102<br>333102L<br>333103L<br>333105D<br>333104B<br>333102A<br>333104<br>333105B<br>333105E<br>333105F<br>333105G<br>333090L	Z3619	

Appendix 3: cohort generation by country, numbers constitute person -years (thousands)

		US	Canada	England	Netherlands[1]	Israel	Taiwan
	**Initial cohort**	2,143	158	108	126	39	134
Filter 1	Exclude if not first AMI admission in the past 365 days	62	10	12	2	4	15
Filter 2	Exclude if missing age or sex	0	0	0	0	0	1
Filter 3	Exclude if age < 66	342	61	35	54	16	57
Filter 4	Exclude if residing out of area	16	0	0	0	0	0
Filter 5	Exclude if <12 months continuous enrollment before admission	115	0	0	0	0	0
Filter 6	Exclude if <12 months continuous enrollment after admission (unless died)	1	0	7	0	0	0
Filter 7	Exclude if 2+ months consecutive MA[2] in 12 months before admission	511	N/A	N/A	N/A	N/A	0
Filter 8	Exclude if 2+ months consecutive MA in 12 months after admission	31	N/A	N/A	N/A	N/A	0
	**Final cohort**	1,064	86	53	70	19	61

1. Person-years are for population in 2013-2017 sample due to data unavailability: 2. MA= Medicare Advantage

Appendix 4: Number of AMI hospitalizations by country and year

		2011	2012	2013	2014	2015	2016	2017	Total
**US**	**STEMI**	42,771	40,065	37,183	36,281	35,238	34,101	32,917	258,556
	**NSTEMI**	118,797	117,267	112,715	112,934	114,590	115,516	113,724	805,543
**Canada**	**STEMI**	3,071	3,021	3,171	3,253	3,293	3,428	3,473	22,710
	**NSTEMI**	8,793	9,234	9,054	9,143	9,092	9,335	9,039	63,690
**England**	**STEMI**	1,655	2,100	2,117	2,142	2,147	2,098	2,056	14,315
	**NSTEMI**	6,613	6,271	5,974	5,545	4,996	4,831	4,851	39,081
**Netherlands**	**STEMI**	na	na	4,301	4,919	5,130	5,125	4,913	24,388
	**NSTEMI**	na	na	7,803	8,568	9,362	9,668	9,954	45,355
**Israel**	**STEMI**	764	779	768	659	652	718	667	5,007
	**NSTEMI**	1,866	1,809	2,002	1,985	2,060	2,198	2,116	14,036
**Taiwan**	**STEMI**	3,886	3,776	3,781	3,503	3,436	3,453	3,316	25,151
	**NSTEMI**	4,094	4,520	4,841	5,114	4,961	5,831	6,134	35,495

Appendix 5: Age and sex standardized rates of hospitalization (hospitalization per 1,000 adults age ≥ 66-years of age per-year) for ST elevation and non -ST elevation myocardial infarction (STEMI and NSTEMI) in the US, Canada, England, Netherlands, Israel and Taiwan (2011 -2017)

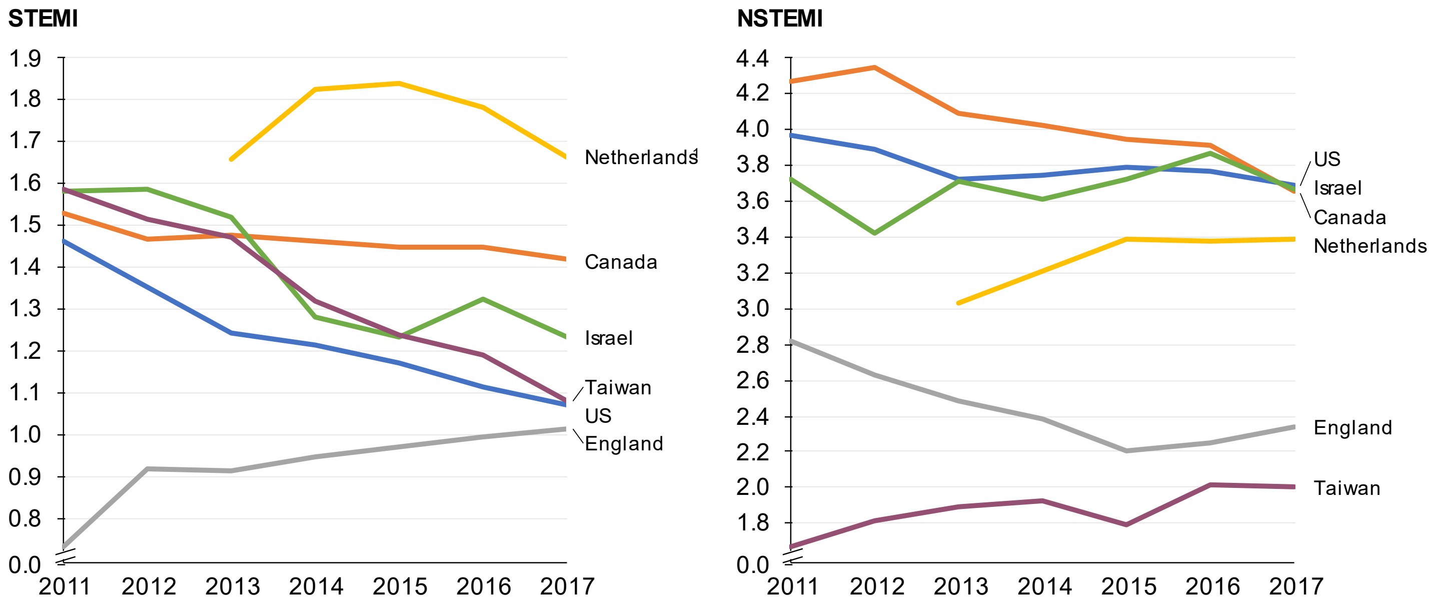

1. Data unavailable for 2011 -12

Appendix 6: Age and sex standardized percentages of patients receiving CABG, PCI and cardiac catheterization (with or without intervention), mean hospital length of stay (LOS), and unadjusted median LOS (with interquartile range [IQR]) for patients hospitalized with STEMI and NSTEMI, 2011-2017

	**Metric**	**STEMI**							**NSTEMI**						
		**2011**	**2012**	**2013**	**2014**	**2015**	**2016**	**2017**	**2011**	**2012**	**2013**	**2014**	**2015**	**2016**	**2017**
**US**	CABG in hospital	7.9%	7.3%	7.1%	7.0%	6.9%	6.5%	6.4%	9.8%	9.8%	10.3%	10.5%	10.6%	11.0%	10.8%
	CABG within 90 days of index admission	9.3%	8.5%	8.4%	8.3%	8.1%	7.6%	7.3%	10.9%	10.9%	11.4%	11.5%	11.6%	12.0%	11.7%
	PCI in hospital	62.9%	65.8%	67.6%	68.9%	70.2%	71.1%	71.8%	29.5%	30.3%	30.8%	31.0%	31.6%	31.8%	31.7%
	PCI within 90 days of admission	63.2%	66.1%	68.0%	69.2%	70.5%	71.7%	73.7%	30.7%	31.4%	31.9%	32.0%	32.7%	33.1%	33.3%
	Cardiac catheterization in hospital	78.4%	80.2%	81.5%	82.4%	83.7%	84.8%	85.8%	58.0%	58.8%	59.8%	60.3%	61.2%	62.0%	62.0%
	Cardiac catheterization within 90 days of admission	78.9%	80.6%	81.8%	82.8%	84.1%	85.2%	86.3%	59.6%	60.3%	61.2%	61.8%	62.5%	63.5%	63.6%
	LOS, mean	5.66	5.44	5.33	5.35	5.25	5.13	5.11	6.47	6.26	6.20	6.21	6.18	6.14	6.08
	LOS, median (IQR)	4 (2-7)	4 (2-6)	3 (2-6)	3 (2-6)	3 (2-6)	3 (2-6)	3 (2-6)	4 (3-8)	4 (3-7)	4 (3-7)	4 (3-7)	4 (3-7)	4 (3-7)	4 (3-7)
**Canada**	CABG in hospital	3.4%	3.0%	3.5%	2.5%	2.8%	3.2%	2.9%	7.2%	7.5%	7.5%	7.8%	8.0%	8.3%	8.3%
	CABG within 90 days of index admission	4.9%	4.2%	4.7%	3.5%	3.8%	4.1%	3.7%	8.8%	9.1%	9.0%	9.2%	9.3%	9.8%	9.6%
	PCI in hospital	66.4%	70.8%	72.8%	73.0%	74.3%	75.2%	78.6%	27.3%	29.2%	31.7%	32.7%	34.8%	36.1%	37.8%
	PCI within 90 days of admission	67.1%	71.2%	73.3%	73.5%	74.5%	75.5%	79.0%	29.0%	30.8%	33.3%	34.0%	36.1%	37.6%	39.4%
	Cardiac catheterization in hospital	78.9%	81.7%	83.7%	83.0%	84.0%	84.7%	87.3%	52.6%	55.2%	58.3%	59.1%	61.0%	63.7%	64.3%
	Cardiac catheterization within 90 days of admission	79.9%	82.2%	84.2%	83.4%	84.4%	85.0%	87.7%	55.5%	57.6%	60.6%	61.2%	63.1%	65.9%	66.3%
Manitoba[i]	LOS, mean	7.2	7.3	6.9	6.7	6.4	6.4	6.5	9.0	8.4	8.0	8.2	7.9	8.0	7.8
	LOS, median (IQR)	6 (4–11)	6 (4–12)	6 (4–11)	6 (4–10)	6 (4–11)	6 (4–10)	6 (4–11)	9 (5–17)	7 (5–14)	7 (4–13)	7 (5–13)	7 (4–14)	7 (4–14)	7 (5–14)
Ontario	LOS, median (IQR)	4 (3-7)	4 (3-7)	4 (2-7)	4 (2-7)	4 (2-6)	3 (2-6)	3 (2-6)	5 (3-9)	5 (3-9)	5 (3-9)	5 (3-9)	5 (3-8)	5 (3-8)	5 (3-8)
**England**	CABG in hospital	1.2%	1.2%	1.7%	1.9%	1.7%	1.6%	1.7%	1.5%	1.6%	1.8%	1.9%	2.1%	2.4%	2.0%
	CABG within 90 days of index admission	3.6%	3.6%	3.8%	4.6%	3.4%	3.1%	3.7%	6.4%	6.0%	6.7%	6.7%	6.7%	6.4%	6.4%
	PCI in hospital	33.6%	33.9%	35.6%	36.9%	33.5%	36.7%	36.9%	18.8%	21.0%	21.7%	22.6%	25.1%	24.3%	26.0%

	PCI within 90 days of admission	37.7%	36.6%	38.3%	39.3%	36.3%	39.2%	40.0%	23.6%	25.0%	26.0%	26.5%	28.7%	28.4%	30.2%
	Cardiac catheterization in hospital	44.4%	43.6%	46.1%	47.6%	42.4%	45.4%	44.0%	40.9%	44.7%	47.5%	47.3%	49.9%	49.2%	51.4%
	Cardiac catheterization within 90 days of admission	50.2%	48.3%	49.8%	51.4%	46.5%	49.0%	48.7%	49.5%	51.3%	53.7%	53.1%	55.4%	55.2%	57.2%
	LOS, mean	6.94	7.02	7.18	6.83	7.05	6.72	6.58	9.60	9.79	9.35	9.22	9.37	9.20	8.62
	LOS, median (IQR)	4 (3-7)	4 (3-8)	4 (2-8)	4 (2-7)	3 (2-7)	4 (2-7)	3 (2-7)	6 (3-11)	6 (3-11)	6 (3-11)	6 (3-11)	6 (3-11)	5 (3-10)	5 (3-10)
**Netherlands**	CABG in hospital	**	**	1.4%	2.2%	2.5%	2.3%	2.3%	**	**	1.5%	2.0%	2.1%	2.4%	2.7%
	CABG within 90 days of index admission	**	**	2.5%	2.8%	3.5%	3.3%	3.0%	**	**	2.3%	2.7%	2.8%	3.2%	3.5%
	PCI in hospital	**	**	32.9%	46.6%	47.9%	48.9%	48.1%	**	**	14.5%	19.7%	21.0%	21.4%	22.0%
	PCI within 90 days of admission	**	**	34.6%	48.9%	50.2%	51.3%	49.8%	**	**	17.0%	22.8%	24.4%	24.6%	24.7%
	Cardiac catheterization in hospital	**	**	39.2%	56.1%	58.2%	59.0%	57.5%	**	**	27.1%	47.1%	50.0%	51.7%	53.3%
	Cardiac catheterization within 90 days of admission	**	**	41.6%	59.0%	60.8%	61.8%	59.5%	**	**	30.6%	50.6%	53.2%	55.0%	56.0%
	LOS, mean	**	**	5.73	5.52	5.51	5.20	4.98	**	**	6.29	6.16	6.08	5.86	5.78
	LOS, median (IQR)	** (**)	** (**)	4 (2-7)	4 (2-6)	4 (2-6)	4 (2-6)	3 (2-6)	** (**)	** (**)	5 (3-8)	5 (3-8)	4 (3-8)	4 (3-7)	4 (2-7)
**Israel**	CABG in hospital	4.8%	3.9%	2.8%	2.8%	2.3%	2.0%	3.1%	5.9%	4.8%	3.9%	3.8%	2.3%	2.9%	2.8%
	CABG within 90 days of index admission	6.5%	6.0%	5.7%	4.6%	3.9%	4.5%	4.7%	7.8%	7.3%	7.1%	6.9%	5.0%	5.7%	5.6%
	PCI in hospital	48.4%	50.0%	54.2%	54.8%	55.6%	59.3%	65.9%	23.6%	23.3%	28.4%	27.8%	32.2%	37.9%	39.2%
	PCI within 90 days of admission	51.0%	52.3%	58.3%	59.6%	61.4%	64.4%	70.4%	27.0%	26.8%	32.0%	32.6%	36.4%	43.5%	45.0%
	Cardiac catheterization in hospital	61.7%	61.9%	63.5%	66.6%	68.5%	70.8%	73.6%	42.7%	41.8%	45.0%	42.9%	48.4%	54.7%	55.9%
	Cardiac catheterization within 90 days of admission	65.2%	64.8%	68.2%	72.6%	74.6%	75.6%	78.4%	48.0%	46.7%	51.0%	49.6%	54.0%	61.0%	62.0%
	LOS, mean	6.81	7.26	6.98	7.29	7.09	8.01	5.98	7.50	7.04	7.19	7.34	6.83	6.62	6.82
	LOS, median	4	4	3	3	3	3	3	5	5	5	5	5	4	4
**Taiwan**	CABG in hospital	4.6%	4.4%	4.8%	5.2%	4.5%	3.4%	2.8%	4.2%	4.2%	4.6%	4.6%	4.3%	3.7%	3.3%
	CABG within 90 days of index admission	5.7%	5.3%	5.4%	6.0%	5.2%	4.1%	3.2%	5.6%	5.6%	5.6%	5.8%	5.2%	4.6%	4.2%
	PCI in hospital	49.4%	54.3%	58.0%	61.4%	65.0%	65.2%	70.2%	36.6%	38.5%	41.1%	44.2%	49.1%	48.0%	50.7%

	PCI within 90 days of admission	51.1%	55.6%	59.1%	62.8%	65.8%	66.0%	70.8%	39.3%	40.8%	43.7%	46.6%	51.1%	49.5%	52.7%
	Cardiac catheterization in hospital	63.8%	66.2%	70.0%	73.4%	75.5%	76.1%	79.0%	55.8%	57.6%	60.8%	63.8%	69.5%	67.3%	68.6%
	Cardiac catheterization within 90 days of admission	66.1%	68.1%	71.7%	74.9%	76.5%	76.9%	79.8%	59.7%	60.8%	63.9%	66.5%	71.4%	69.2%	70.5%
	LOS, mean	9.96	10.29	9.83	9.53	9.68	9.19	8.54	11.60	11.55	11.03	10.54	9.64	10.27	9.54
	LOS, median (IQR)	6 (3-11)	6 (3-11)	5 (3-10)	5 (3-10)	5 (3-10)	5 (3-9)	5 (3-9)	7 (4-14)	7 (4-13)	7 (4-13)	7 (4-12)	6 (4-10)	6 (4-11)	6 (4-11)

** Data unavailable

[1] Manitoba and Ontario reported separately for median LOS

Appendix 7: Age and sex standardized mortality and readmissions after hospitalization for STEMI and NSTEMI by country, 2011-2017

Country	Metric	STEMI							NSTEMI**						
		2011	2012	2013	2014	2015	2016	2017	2011	2012	2013	2014	2015	2016	2017
**US**	**Death within 30 days**	19.8%	19.6%	19.3%	19.4%	18.8%	19.1%	18.8%	12.3%	12.3%	12.3%	12.4%	12.0%	11.7%	11.8%
	**Death within one year**	30.3%	29.6%	28.7%	29.0%	28.1%	28.3%	27.8%	30.6%	30.5%	29.4%	30.2%	29.3%	29.1%	29.3%
	**Readmission within 30 days**	14.3%	13.1%	12.7%	12.0%	12.3%	12.0%	12.2%	18.3%	17.3%	16.5%	16.3%	16.1%	16.1%	16.1%
**Canada**	**Death within 30 days**	15.8%	17.8%	16.9%	16.9%	17.7%	16.4%	15.9%	13.1%	11.8%	11.4%	11.4%	11.4%	10.1%	10.6%
	**Death within one year**	24.1%	24.6%	24.4%	25.0%	25.6%	25.0%	23.0%	27.8%	26.6%	25.8%	25.2%	25.1%	24.1%	24.3%
	**Readmission within 30 days**	13.7%	14.9%	15.0%	14.3%	13.3%	13.7%	14.1%	16.4%	16.6%	16.8%	15.8%	15.9%	15.3%	15.1%
**England**	**Death within 30 days**	10.3%	9.9%	10.8%	8.9%	12.6%	14.2%	13.3%	10.0%	9.9%	10.4%	10.2%	9.7%	9.7%	8.9%
	**Death within one year**	16.7%	16.5%	16.7%	15.9%	20.0%	20.8%	19.1%	20.7%	21.6%	20.8%	21.3%	21.1%	20.8%	21.8%
	**Readmission within 30 days**	26.3%	26.6%	24.6%	26.1%	25.3%	25.1%	23.1%	35.6%	35.2%	36.9%	35.1%	35.5%	34.7%	36.2%
**Netherlands**	**Death within 30 days**	**	**	15.0%	13.4%	13.9%	14.1%	12.7%	**	**	9.9%	9.0%	8.5%	7.7%	7.6%
	**Death within one year**	**	**	21.7%	19.5%	20.1%	19.7%	18.9%	**	**	20.7%	20.2%	19.2%	17.1%	17.4%
	**Readmission within 30 days**	**	**	22.3%	19.1%	22.1%	21.1%	19.2%	**	**	26.7%	23.6%	26.2%	25.0%	23.7%
**Israel**	**Death within 30 days**	10.1%	10.5%	12.6%	14.5%	12.5%	8.5%	14.1%	11.0%	10.7%	10.7%	10.6%	11.9%	9.3%	10.4%
	**Death within one year**	19.4%	21.9%	22.5%	23.9%	20.9%	20.8%	24.3%	27.3%	27.7%	27.5%	27.4%	26.0%	24.7%	25.1%
	**Readmission within 30 days**	19.5%	19.7%	19.9%	19.4%	19.9%	20.7%	19.0%	23.1%	22.7%	23.6%	21.6%	22.9%	19.5%	22.9%
**Taiwan**	**Death within 30 days**	25.2%	24.1%	23.8%	21.8%	22.5%	22.2%	22.2%	14.7%	14.6%	13.7%	13.8%	13.6%	14.0%	14.0%
	**Death within one year**	39.2%	37.5%	36.5%	34.0%	35.8%	33.4%	32.3%	35.1%	36.3%	34.0%	33.2%	32.4%	32.2%	33.0%
	**Readmission within 30 days**	13.7%	12.9%	12.1%	14.6%	13.5%	12.4%	11.7%	17.8%	16.8%	17.9%	17.1%	16.9%	16.8%	16.5%

** Data unavailable
